# Assessment of head gunshot wounds by means of post-mortem computed tomography in exhumed anonymous cadaver

**DOI:** 10.1259/bjrcr.20150304

**Published:** 2016-11-02

**Authors:** Artur Wojciechowski, Marcin Fudalej, Paweł Skowronek

**Affiliations:** ^1^Radiology Department, St George’s University Hospitals NHS Foundation Trust, London, United Kingdom; ^2^Forensic Medicine Department, Medical University of Warsaw, Warsaw, Poland; ^3^Orthopaedics and Traumatology Department, Jan Kochanowski University, Kielce, Poland

## Abstract

Autopsy of corpses with advanced post-mortem changes is the most challenging aspect of medico-legal activities. In many cases, owing to soft tissue decomposition, making a final diagnosis as to the mechanism and cause of death is very difficult, and sometimes impossible (Carcione P, Argo G, Pincone D, Zgo S, Scopelliti L, Sortino C, Procaccianti P. Role of MCT vitropsy in evaluation of burned bodies and its comparison with traditional autopsy. Poster No.: C-1156, ECR 2014, Scientific exhibit). In such cases, the diagnostic process can be supported by post-mortem CT imaging. Post-mortem multislice CT imaging used in the field of forensic medicine is widely reported to be a good method for visualizing injuries and natural pathologies; however, only a limited number of forensic departments use this method in everyday practice. This method enables accurate assessment of bony injuries (fracture type, degree of bone displacement); has the ability to detect radiopaque foreign bodies, most frequently fragments of bullets; and in some cases enables soft tissue delineation (Hardy K. CT autopsy. *Radiology Today* 2008; 9: 20. Available from: http://www.radiologytoday.net/archive/rt01282008p20.shtml). In cadavers with advanced post-mortem changes, it is extremely difficult to retrieve the whole bullet or its parts. Owing to decomposition and reduced cohesion of the tissues, standard autopsy preparation techniques are impossible to perform. Post-mortem changes may also cause displacement of the bullet within the body in the long term, as well as at the time of transport following exhumation (Maiese A, Gitto L, De Matteis A, Panebianco V, Bolino G. Post mortem computed tomography: useful or unnecessary in gunshot wounds deaths? Two case reports. *Leg Med (Tokyo)* 2014; 16: 357–63). It is therefore important to perform post-mortem CT imaging directly after extraction of corpses in a similar position to how the dead body was exhumed. Interpretation of the images requires cooperation of forensic medicine specialists and radiologists to correlate radiological findings with autopsy.

## Background

The common feature of all gunshot wounds is the presence of damage to the soft tissues at the site of entry along with the presence of a rim of bruising at the vicinity of the entry wound, which is also called contusion haematoma. Depending on the type and energy of the bullet, and the distance from which the round was fired, additional signs may also be visible. For example, wounds inflicted by close range shots are characterized by the presence of gunpowder tattoo—particles of gunpowder embedded in the skin and skin burn due to hot exhaust gases from the barrel. Unfortunately, these changes are not clearly seen in the soft tissues undergoing post-mortem decomposition and change. Even potentially fixative changes such as mummification or fat-wax transformation can obscure the features of an entry wound. Bullet calibre and length of the gun barrel affect the initial speed of the projectile and its effective range. The ability to penetrate the tissue depends on the material the bullet is made of, flight trajectory and angle of penetration. Unjacketed, relatively soft bullets passing through the tissues are readily deformed—flattened or distorted. Hitting bony structures may change the trajectory of a bullet—deflect it from the straight line or ricochet. Jacketed bullets fired from rifles, some hand guns and automatic guns have significantly higher barrel exit velocity, and thus energy, than unjacketed bullets. The external cover makes them less prone to shape change and distortion during the initial contact with soft tissues. Contact with bone may lead to burst fracture, with minimal change in the trajectory of the bullet. Shotgun shells contain multiple small beads or lead slugs that exit the barrel at a lower speed; however, the large number of small projectiles can cause extensive damage to the body, particularly with close range gunshots.^1^

In the absence of an exit wound, also called a blind shot, it is important to find the whole bullet or its smaller fragments. In cadavers with advanced post-mortem changes, it is extremely difficult to retrieve the whole bullet or its parts. Owing to decomposition and reduced cohesion of the tissues, standard autopsy preparation techniques are impossible to perform. Post-mortem changes may also lead to displacement of the bullet within the body in the long term, as well as at the time of transport following exhumation. It is therefore important to perform post-mortem CT (PMCT) directly after extraction of corpses in a similar position to how the dead body was exhumed.

## Case presentation

The exhumed remains of our case were taken to the Department of Forensic Medicine. Following preliminary assessment of the corpse, of which the head had separated in a natural way and was covered with soil and plant roots, and the trunk was encased in concrete, a decision was made to perform PMCT of the head prior to autopsy to rule out potential lethal injuries. There was a suspicion of death following a gunshot wound. PMCT was performed with a 16-slice multidetector helical CT scanner (Toshiba, Otawara, Japan), 1 mm slice collimation and secondary image reconstruction with soft tissue and bone windows. The aim of the study was to determine the nature of possible injuries and document the original location of possible foreign bodies; identify the number and type of bullets, number of inlet and outlet wounds and degree of damage to the bony structures, especially the facial bones; and possibly evaluate the soft tissues. It was possible that some of the bullets’ fragments could be embedded deep within the bony structures of the base of the skull and some could also be located outside the body (*e.g.* in the surrounding soil). The latter can be displaced during autopsy. Images were reviewed with the IntelliSpace Portal software (Philips, Eindhoven, Netheralnds) to perform multiplanar and volume reconstructions. Following the CT examination, a routine post-mortem examination of the body was performed in the autopsy room to assess macroscopic tissue damage and retrieve metallic foreign objects. Evaluation of soft tissues was much more difficult, and in many regions impossible, because of significant decomposition and destruction. We also believed that technique of tissue preparation and separation could result in the displacement of small fragments of bones and metallic bullets.

## Imaging and autopsy findings

**Figure 1.**
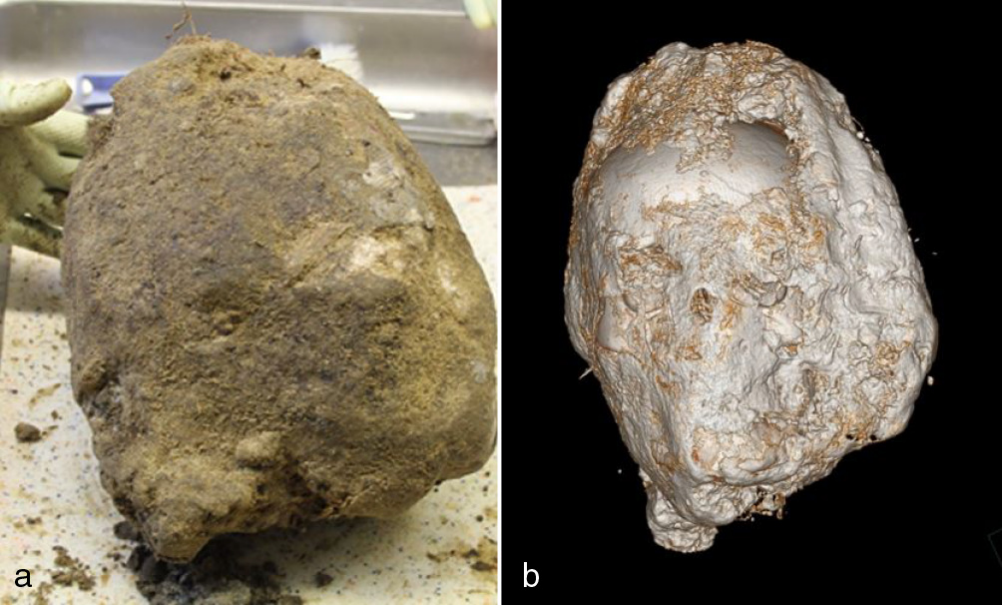
(a) Extracted head of the exhumed cadaver covered with soil. (b) Three-dimensional reconstruction of the specimen following post-mortem CT.

PMCT examination of the head identified two bullet entry wounds—one within the left mastoid process and the other within the squamous part of the left temporal bone. No exit wounds were identified. The examination revealed the presence of two dominant-sized metallic foreign bodies located within the skull in the parietotemporal area near the inlet wound. Furthermore, a fracture within the squamous part of the right temporal bone on the opposite side of the entry wound was noted with small bony fragments displaced slightly outward (a “blow-out fracture”) and no clear exit wound, which indicated that the damage was caused by the impact of the projectile and that the energy of the projectile was too low to fully penetrate the bone and create an exit wound. In addition, there were several small metallic elements within the displaced, and significantly decomposed and contracted brain tissue located in the lower most part of the exhumed skull on the left side. The cadaver was buried in the left lateral position. Following exposure of the skull, only the dural reflexions could be identified: the falx cerebri, tentorium cerebelli and remaining structures had been damaged by the decomposition process (Figure 1a,b).

The autopsy revealed advanced post-mortem soft tissue changes with partial skeletonization and significant necrolysis. It confirmed the presence of two gunshot entry wounds on the left side of the head (one on the left mastoid process and the other on the squamous part of the left temporal bone). Two deformed bullets were found surrounded by necrotically transformed brain tissue. One of the bullets was significantly deformed, indicating that it had penetrated through the hard part of the pyramid of the temporal bone. The second bullet was deformed to a lesser degree and most likely had penetrated, as well as caused damage to, the squamous part of the temporal bone on the contralateral side, leading to a blow-out fracture. Both the bullets were retained within the skull with no exit wounds present.

**Figure 2.**
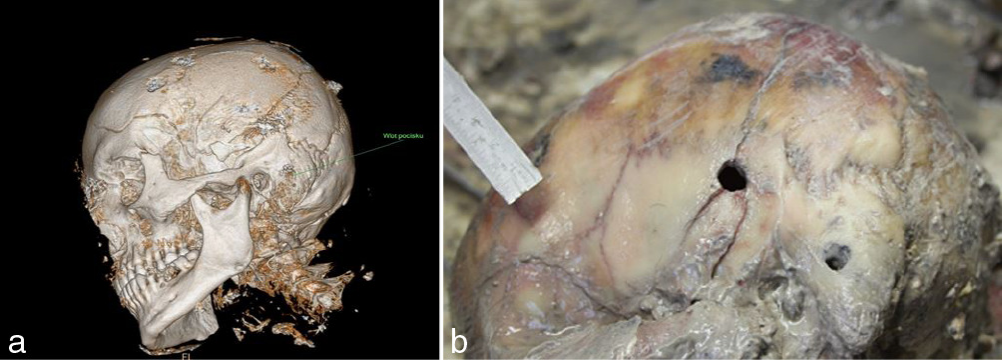
(a) Two entry wounds on the left side of the head seen on post-mortem CT reconstruction. (b) Photograph taken during autopsy.

**Figure 3.**
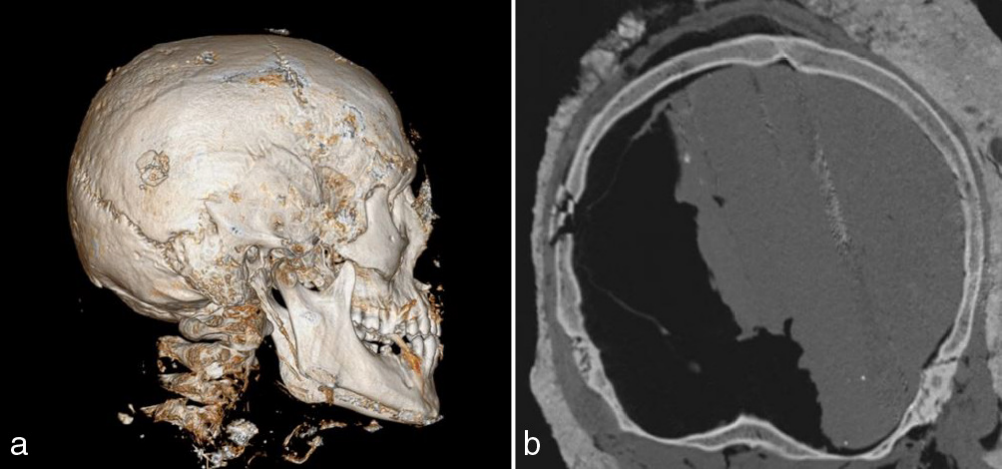
(a, b) Blow-out fracture showing few slightly displaced bony fragments within the squamous part of the right temporal bone.

**Figure 4.**
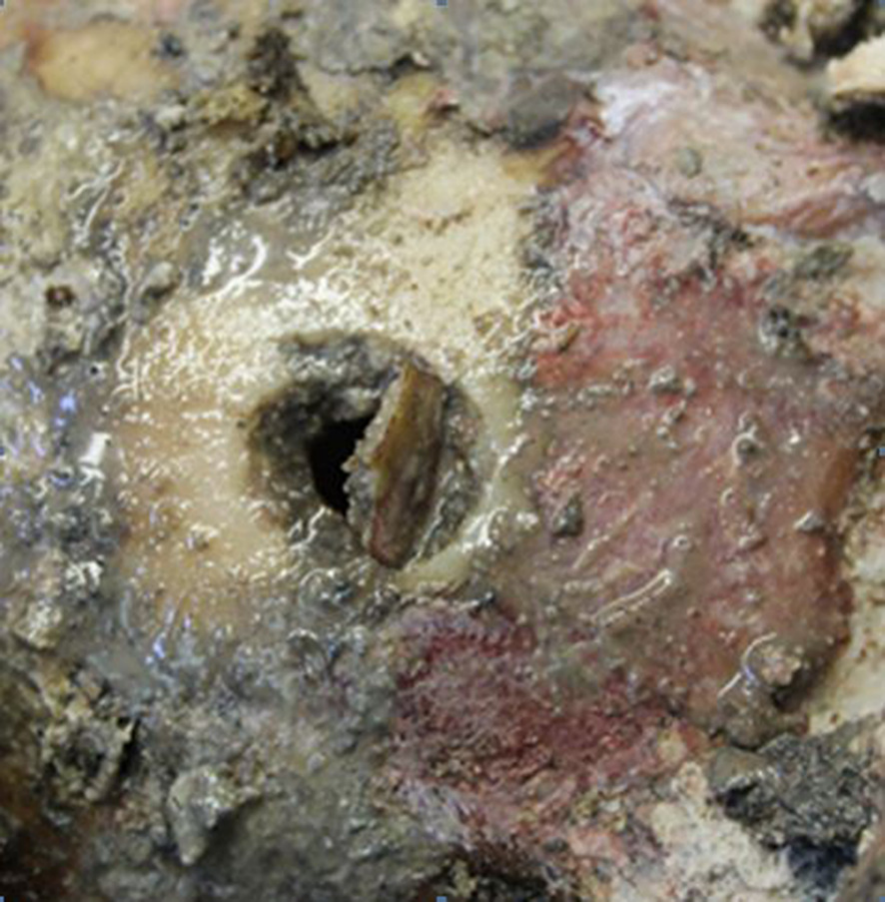
Blow-out fracture in the area opposite to the entry wounds with overlying bony fragment that was displaced during the autopsy.

In addition, autopsy revealed a comminuted fracture, with no significant displacement of the right zygomatic arch that was not clearly visible on CT examination (Figures 2–4).

The post-mortem examination also involved an assessment of the trunk and extremities. It showed typical post-mortem changes and some loss of peripheral small bones with no evidence of mechanical damage of the examined bones and ligamentous structures.

Advanced post-mortem changes may significantly reduce the diagnostic value of autopsy in determining the cause of death. However, in the described case, the presence of injuries typical for gunshot wounds with bullets retained within the skull indicated that the death of the person had occurred as a result of the gunshot injuries to the head.

## Discussion

CT imaging has been successfully used in forensic medicine in many centres worldwide for several years.^2,3^ For a long time, the only diagnostic method available at the forensic medicine departments were plain X-rays, which were used for assessing the bony structures and searching for metallic foreign objects in cadavers.^4^ The main drawback of this diagnostic method is the principle of image formation, in which a three-dimensional (3D) object is projected on to a two-dimensional X-ray film or digital panel detector. X-ray exposures taken in two orthogonal projections enable more precise location of the foreign objects within the cadaver body. It is recommended to use digital radiographic systems that allow adjustment of brightness and contrast of images, as well as automatic exposure control to optimize exposure conditions. In CT, data acquisition is performed at the time of movement of the X-ray tube around the patient’s body by a detector located opposite the lamp; during the examination, the table on which the patient is lying moves at a constant speed. Depending on the slice thickness, the table movement speed and the X-ray tube rotation speed, the whole body may be examined with partially overlapping slices, which are more suitable for better secondary reconstruction protocols.

Protocols of CT examinations performed in the forensic medicine department setting differ from those for living patients. In living patients, the largest scanned length involves the trunk in trauma cases, or the abdomen and lower limbs in arterial runoff protocols. Post-m-rtem CT examination should be berformed with thin slices covering the whole torso and extremities to allow subsequent good quality 3D and MPR reconstructions.^5^ Radiation dose is not a concern in these settings. The most important technical parameters of a scanner for the forensic medicine department are maximum table range of motion, X-ray tube heat capacity and gantry diameter. In some cases, the study may have to be performed on a cadaver in clothes, covered with soil, concrete, or even inside containers of various types such as coffins, suitcases or chests. Most of the bodies are discovered in unusual positions secondary to the environment that they were buried in, such as squeezed by the pressure of the overlying soil or in-growing plants, and pre-mortem restriction of the limbs. This would require modification of the standard scanning protocols such as increasing the X-ray tube current to increase the number of photons passing through the object, extending the scan range, and use of appropriate filters and reconstruction protocols, particularly for imaging the bony parts. Unfortunately, decomposition makes soft tissues extremely difficult or impossible to assess.^6^ Reviewing the images on a workstation and trying to perform 3D reconstructions can be challenging in terms of manual removal of soil and stones adjacent to the imaged structures. In some cases, the surrounding material may also contain important evidence, such as a bullet or parts thereof. It is a time-consuming process.

Analysis and interpretation of PMCT of significantly changed cadavers requires experience and co-operation of radiology and forensic specialists.

## Learning points

1. CT is a valuable tool to supplement post-mortem examination and enables precise evaluation of the structures that are difficult to access during a routine autopsy.
2. Performing PMCT imaging of grossly decomposed and metamorphosed corpses can help, and sometimes even allow reconstruction of the mechanism of injury and collect suitable evidence.
3. PMCT is a non-invasive method that allows complete assessment of the severity of the injuries in cadavers. It is particularly useful for 3D reconstruction of bony structures, allowing visualization of fractures and displacement that may provide clues to the injury mechanism.
4. PMCT allows for precisely locating metallic foreign objects such as bullets or small metallic fragments.
5. Protocols of CT examinations performed in the forensic medicine department setting differ from those for living patients, and image interpretation requires experience and knowledge of the necrochemical changes in the human body.
6. In PMCT examinations, scanning time and radiation dose does not constitute a restriction for the method.

## Consent

The authors were unable to obtain consent for in this case as it was an anonymous male cadaver exhumed a few years after traumatic death.
